# On the Endocircular Li@C_16_ System

**DOI:** 10.3389/fchem.2022.813563

**Published:** 2022-02-04

**Authors:** Yi-Fan Yang, Lorenz S. Cederbaum

**Affiliations:** Theoretical Chemistry, Institute of Physical Chemistry, University of Heidelberg, Heidelberg, Germany

**Keywords:** charge-separated, van der vaals forces, noncovalant interactions, electronic structure, ab intio calculation

## Abstract

The endocircular Li@C_16_ is a promising system as it can form both a charge-separated donor-acceptor complex and a non-charge-separated van der waals complex. By employing the state-of-the-art equation-of-motion coupled-cluster method, our study shows that the carbon ring of this system possesses high flexibility and may undertake large distortions. Due to the intricate interaction between the guest Li^+^ cation and the negatively charged ring, this system can form several isomers possessing different ground states. The interesting electronic structure properties indicate its applicability as a catalyst candidate in the future.

## I Introduction

Carbon allotropes, e.g., graphene (Xiang et al., 2012), fullerene (Yan et al., 2016), carbon nanotubes (Serp et al., 2003) etc., are playing important roles in the field of catalysis. One of the reasons for those successful catalytic applications is the non-covalent functionalization between carbon allotropes and reactants (Umadevi et al., 2014; Georgakilas et al., 2016). Here, we briefly mention common examples of non-covalent interactions, e.g., interactions forming electron donor-acceptor complex (D’Souza and Ito, 2009; Lim et al., 2016), and van der Waals interactions (Sun et al., 2021).

Carbon allotropes doped with guest atoms can improve their catalystic properties and have been successfully applied in many fields, such as artificial photosynthesis (Rudolf et al., 2016), N_2_ activation (Kumar et al., 2016), electrochemical water splitting (Singh et al., 2016), and electrochemical oxygen reduction (Guo et al., 2020) etc., The improvement of the catalytic properties is in many cases due to the roles played by guest atoms in charge-separated donor-acceptor complexes (Sherigara et al., 2003; Hu et al., 2017), or in stronger van der waals interactions which are enhanced by the guest atom (Biroju et al., 2017; Bawari et al., 2019). It is noteworthy that donor-acceptor charge-separated states may have substantial potential for applications in the field of catalysis (Meng et al., 2020) and as shown in a theoretical study, the change of charge distribution is a reason for enhancing the catalytic property of donor-acceptor charge-separated systems (He et al., 2017).

It is a well-accepted idea that alkali guest atoms can easily form charge-separated donor-acceptor systems with carbon allotropes (Ohkubo et al., 2012; Wang et al., 2012; Yang et al., 2019a). Recently, our group discovered that carbon allotropes with alkali guest atoms can form non-charge-separated states, like caged-electron states in endohedral fullerenes (Yang et al., 2019b), split-electron states in endohedral fullerenes (Yang and Cederbaum, 2021a), and encircled-electron states in carbon rings (Yang and Cederbaum, 2021b). By electronically exciting an endocircular Li carbon ring system, the interaction between the carbon rings and the Li guest atom can shift from donor-acceptor essentially electrostatic interaction to van der waals interaction and vise versa (Yang and Cederbaum, 2021b). This unique property of the Li atom points to potential applications as a catalyst.

With different interaction effects between Li and the carbon rings in different electronic states, the system would prefer different positions of the guest Li. Our calculations on the neutral Li@C_12_ showed that it possesses D_6*h*_ symmetry in its electronic ground state, indicating that finding the correct geometry of this endocircular system is still a challenge for the popular density functional theory (DFT), which predicts an off-center geometry of the neutral Li@C_12_ (Yang and Cederbaum, 2021b). Hence it is necessary to optimize the Li carbon ring geometries by employing state-of-the-art high level *ab initio* methods, to obtain reliable geometries (Yang and Cederbaum, 2021b). Due to the considerable amount of computational resources required by high-level methods, one can only optimize relatively small endocircular Li carbon ring systems. In this paper, we focus on computing accurately the geometries and electronic structures of next smallest example, i.e., neutral Li@C_16_, which is expected to exhibit an off-center endocircular structure (Yang and Cederbaum, 2021b).

## II Computational Details

The C_16_ carbon ring is formed by short and long bonds, which we just call for simplicity triple and single bonds, repectively. Acoording to our calculations there are three structures of planar off-center neutral Li@C_16_ with different off-center Li positions. To lower the total energy of the system, Li can move from the center of the ring towards one of the single C-C bonds, one of the triple C-C bonds, or towards one of the C atoms. The first two structures are found to be in C_2*v*_ symmetry and the last one is in C_*s*_ symmetry.

By employing the state-of-the-art equation-of-motion coupled-cluster singles and doubles method for electron affinities (EA-EOM-CCSD) (Nooijen and Bartlett, 1995) with the CFOUR code (Matthews et al., 2020), we optimized the geometries of ground states of neutral Li@C_16_ isomers in C_2*v*_ symmetry. The basis sets used are Dunning correlation-consistent triple-zeta basis sets (cc-pVTZ). (Dunning, 1989; Prascher et al., 2011).

We employ the corresponding cationic wavefunction of Li^+^@C_16_ as the reference wavefunction of the EA-EOM-CCSD calculations, since these cationic closed-shell systems are suitable reference states for describing the binding of an additional electron and thus for the calculations of the ground and excited states of the neutral Li@C_16_ system. In this paper, the electron binding energy (EBE) is referred to as the energy gain of the neutral Li@C_16_ systems obtained by adding an electron to its cation in the respective geometry of the neutral system. This electron is addressed as the excess electron. At the optimized geometries, we also calculated the single point energies of the low-lying states of neutral Li@C_16_ at EA-EOM-CCSD/cc-pVTZ level. The core orbitals of C and Li atoms were not frozen in all the EA-EOM-CCSD studies.

Due to the high computational cost involved, we did not carry out the geometry optimization of neutral Li@C_16_ in C_*s*_ symmetry with the EA-EOM-CCSD method. Instead, we employed the density functional theory. The performance of various DFT functionals for carbon rings has been discussed in several studies (Jin et al., 2015; Kaiser et al., 2019). Most DFT functionals have been found to be unreliable in treating carbon ring systems. The *ω*B97XD (Chai and Head-Gordon, 2008) functional has been found to perform well predicting the correct polyynic geometry of the ground state of cyclo [18] carbon ring system (Baryshnikov et al., 2019; Liu et al., 2020). It has been just now applied to another endocircular lithium carbon ring system by other researchers (Liu et al., 1872022). Accordingly, we employed here *ω*B97XD with the cc-pVTZ basis set, using the Gaussian 09 package (Frisch et al., 2009). For completeness we also optimized the C_2*v*_ structures by employing this DFT method and compare them with those obtained via the EA-EOM-CCSD method.

## III Results and Discussion

### A *The Equilibrium Geometries of Li@C*_16_

Planar even number carbon rings C_*n*_ have attracted considerable attentions (Prinzbach et al., 2006; Jin et al., 2015; Baryshnikov et al., 2019; Kaiser et al., 2019; Liu et al., 2020). These species can be divided into two classes, i.e., C_4*m*+2_ and C_4*m*_, where m is a natural number. The former ones may possess aromaticity property, resulting in cumulenic geometry formed by double C-C bond only, since they satisfy Hückel’s rule (Arulmozhiraja and Ohno, 2008; Heaton-Burgess and Yang, 2010). The latter ones are polyynic and do not satisfy this rule, forming alternating single and triple C-C bonds. As a well investigated example, theoretical studies on neutral C_20_ show that its geometry is polyynic (Jin et al., 2015; Yang and Cederbaum, 2020).

**FIGURE 1:**
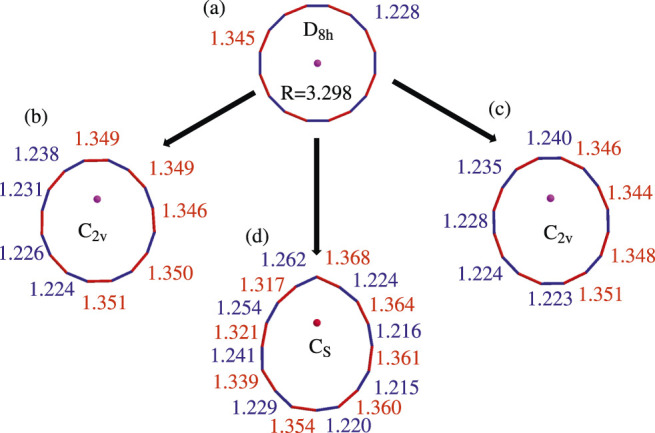
Computed optimized geometries of the ground state of neutral Li@C_**16**_. **(A)** In D_**8*h***_ symmetry (at EA-EOM-CCSD/cc-pVTZ level). **(B)** In C_**2*v***_ symmetry with off-center Li^**+**^ towards the single C-C bond (at EA-EOM-CCSD/cc-pVTZ level). **(C)** In C_**2*v***_ symmetry with off-center Li^**+**^ towards the triple C-C bond (at EA-EOM-CCSD/cc-pVTZ level). **(D)** In C_***s***_ symmetry with off-center Li^**+**^ towards one carbon atom (at ***ω*** B97XD/cc-pVTZ level). The bonds colored in red and blue are the single and triple C-C bonds, respectively.

There are amazingly few papers focusing on endocircular carbon rings. In our previous study (Yang and Cederbaum, 2021b), we have optimized the electronic ground state equilibrium geometry of Li@C_16_ in D_8*h*_ symmetry. As shown in Figure 1A, the equilibrium structure is polyynic with alternating single and triple bonds. As the electronic ground state possesses a charge-separated nature, where Li appears as a cation and the carbon ring is negatively charged, it is likely that the Li is off-center in this system in order to minimize the energy. Indeed, the EA-EOM-CCSD computations showed that at D_8*h*_, where the Li is at the center of the ring, the system possesses imaginary frequencies, indicating that structures with in-plane off-center Li^+^ cation are energy favored. However, a reliable ground state geometry of Li@C_16_ with an off-center guest Li^+^ cation, which certainly distorts the C_16_ ring is still unknown.

### A.1 Results

According to our extended search, there are three possible structures of off-center neutral Li@C_16_, two in C_2*v*_ symmetry and one in C_*s*_ symmetry. The two structures in C_2*v*_ symmetry differ in the off-center position of the Li^+^ cation, see structures (B) and (C) in Figure 1. Their Li^+^ cations moved from the center towards a single and a triple bond, respectively. Differently, the Li^+^ cation of structure in C_*s*_ symmetry moved from the center towards a carbon atom as shown (D) in Figure 1.

In contrast to the highly symmetric structure (A) where all triple bonds (colored in blue in Figure 1) and all single bonds (colored in red in Figure 1) have the same length, this is clearly not the case for the other structures. In particular, in structure (B) the bond lengths of the four triple C-C bonds which are closer to the off-center Li^+^ cation are longer, while the other four triple C-C bonds are shorter than their counterparts in structure (A). In contrast, nearly all the single C-C bonds in structure (B) are longer than their counterparts in structure (A).

For the second C_2*v*_ geometry seen in structure (C), one can see that the triple C-C bonds have undergone similar changes in comparison to structure (A). The bond lengths of the triple C-C bonds closer to the off-center Li^+^ cation are elongated while the bond lengths of the single C-C bonds in particular those closer to the Li^+^ (upper part of Figure 1C) became considerably longer than their counterparts in the highly symmetric structure (A).

The distortion of the carbon ring of structure (D) which possesses C_*s*_ symmetry is more significant than in the C_2*v*_ structures (B) and (C). In particular, the uppermost carbon atom in Figure 1D is pushed out such that the upper five carbon atoms nearly form a triangular shape. In spite of the strong changes the ring experienced in (D) compared to the highly symmetric structure (A), it is noteworthy that the various bonds are still alternating and can be classified in shorter and longer (or triple and single) bonds. In the next subsection we attempt to quantify the distortions of the C_2*v*_ and C_*s*_ structures.

The coordinates of all optimized geometries computed in this paper by EA-EOM-CCSD and DFT are shown in Supplementary Tables S1, S2, respectively. We have also computed the vibrational frequencies at EA-EOM-CCSD level for structures (a), (b) and (c) and at DFT level for all the four structures. The results are collected in Supplementary Tables S3, S4, respectively. Based on the frequency calculations, one finds that the structures (a) and (b) are saddle points, while structures (c) and (d) are minima on the potential energy surface.

### A.2 Discussion and Comparison Between Equation-of-Motion Coupled-Cluster and Density Functional Theory

In the structures (b), (c), and (d) the Li^+^ cation is off-center and the carbon ring is distorted. To better understand the differences of the distortion in the various structures, we would like to introduce simple quantities which quantify the situation and compare the results for the structures computed by the EA-EOM-CCSD and DFT methods.

We investigate two quantities. The first one is the distance between the Li^+^ ion and the center of the carbon ring. As the center we take the center of mass of the ring (without Li) and as the masses are all the same and the ring is planar, we simply have

$$\begin{array}{c} x_{center}=\frac{\sum x_{i}}{N},\\ y_{center}=\frac{\sum y_{i}}{N}, \end{array}$$ (1)

where (x_*i*_, y_*i*_) are the coordinates of *i*th carbon atom in the plane and N is the number of carbon atoms. The coordinates of center of mass which we obtained are shown in Supplementary Tables S1, S2. With these coordinates of the center of the ring, one can calculate the distance R_*Li*−*center*_ between the Li^+^ cation and the center of the ring.

Apart from the Li^+^ cation which is shifted off-center as described by R_*Li*−*center*_, we investigate a second measure which describes the distortion of the carbon ring. For that, we calculate the standard deviation *σ*_*r*_ of the carbon-center distances:

$$\sigma_{r}=\sqrt{\frac{\sum_{i=1}^{N}{\left(r_{i}-R\right)}^{2}}{N}},$$ (2)

where N is the number of carbon atoms in the system and r_*i*_ and R are the carbon-center distance of *i*th C atom and the average carbon-center distance, respectively. The results for all the four structures are shown in Table 1.

**TABLE 1:** The distance R_*Li*−*center*_ between the Li^+^ cation and the center of the carbon ring, and the standard deviation *σ*_*r*_ of the distances of the carbon atoms and the ring center of the Li@C_16_ systems studied here. These quantities serve as a measure for the distortion of the structure considered. It is clearly seen that the distortion is growing along the structures (A), (B), (C), and (D) and that it is larger if the geometry is optimized on the EA-EOM-CCSD level of theory. The coordinates used are those of the optimized geometries at both EA-EOM-CCSD and DFT level of theory, see text. The data of struture (A) are taken from (Yang and Cederbaum, 2021b). B. The singly occupied *natural orbitals of Li@C*_16_ *in different geometries*.

	Structure (A)	Structure (B)	Structure (C)	Structure (D)
R_*Li*−*center*_ at EA-EOM-CCSD level (Å)	0.000	1.367	1.385	–
*σ*_*r*_ at EA-EOM-CCSD level (Å)	0.000	0.114	0.130	–
R_*Li*−*center*_ at DFT level (Å)	0.000	1.237	1.244	1.263
*σ*_*r*_ at DFT level (Å)	0.000	0.112	0.117	0.236

As one can see from Table 1, the overall distortion is growing along the structures (A), (B), (C), and (D) and this applies to both the shift of the Li^+^ cation away from the center and the distortion of the carbon ring itself. As mentioned above, optimized geometries are available for all structures on the DFT level and, except for the structure (D) which is of C_*s*_ symmetry, also on the EA-EOM-CCSD level of theory. For the two structures (B) and (C) of C_2*v*_ symmetry the distortion is clearly larger if the geometry is optimized on the EA-EOM-CCSD level of theory.

The structure (D) is particularly intersting. Compared to the two structures in C_2*v*_ symmetry at DFT level, the structure (D) in C_*s*_ symmetry possesses a slightlier larger R_*Li*−*center*_ but a much larger *σ*_*r*_ than all the other systems. This larger *σ*_*r*_ is the result of narrowing the ring on one side and elongating it on the other (see Figure 1). We will further illuminate the uniqueness of the structures in the following section.

### B The Singly Occupied Natural Orbitals of Li@C_16_ in Different Geometries

**FIGURE 2:**
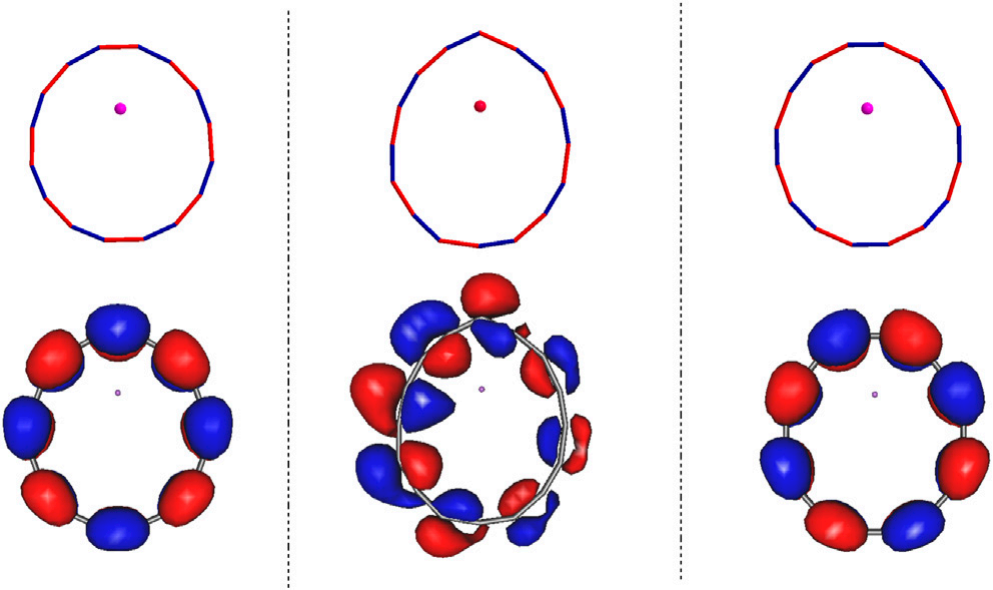
The images of the singly occupied natural orbitals of the ground states of the off-center Li@C_**16**_ structures. The left and right panels show the SONOs for the C_**2*v***_ structure (b) and (c) of Figure 1 where the Li^**+**^ is closer to the single and the triple C-C bond, respectively. The middle panel relates to the C_***s***_ structure (d) of Panel 1, where the Li^**+**^ points to the top C atom. The surfaces shown enclose 80 ***%*** of the electron density.

In all the structures discussed above the Li has donated an electron to the carbon ring resulting in ionic bonding. The resulting singly occupied natural orbital (SONO) describes this excess electron residing on the ring in the presence of its interaction with all the other electrons. The SONOs of the stationary states of Li@C_16_ on the ground electronic potential energy surface along with the corresponding geometries are shown in Figure 2. The left, right, and the middle panels correspond to the structures (b), (c) and (d), respectively. It is particularly relevant to notice that the SONOs of both the C_2*v*_ structures (b) and (c) as well as that of the highly symmetric structure (a) [see (Yang and Cederbaum, 2021b)] are vertical to the plane of the carbon ring, i.e., they form an antibonding pi-electron arrangement. The irreducible representations of these orbitals are B_2_ and A_2_, respectively. The reason for the different natural orbitals of structures of (b) and (c) is clear: These orbitals reflect the different relative positions between the electronic off-center Li^+^ cation and the electron cloud. Due the charge-separated nature of the electronic ground energy surface of Li@C_16_, electrostatic effects play an important role in the stability of this system and their impact on the ring is strongly affected by the position of Li^+^ cation.

The SONO of structure (d) is of interest by itself. As shown in our previous study (Yang and Cederbaum, 2021b), the lowest two charge-separated states of Li@C_*n*_ (*n* = 4 m) are quasi-degenerate and different in symmetry. These two states are gerade and ungerade with respect to reflection through the carbon ring plane. The ungerade state possesses a SONO vertical to the carbon ring plane, while the natural orbital of gerade state is in plane. As we can see in Figure 2, the ungerade state is the ground state of structures in C_2*v*_ symmetry, which is similar to the counterpart in D_8*h*_ symmetry (Yang and Cederbaum, 2021b). In contrast to the C_2*v*_ structures, the ground state of Li@C_16_ in C_*s*_ is gerade with respect to reflection through the carbon ring plane, i.e., the SONO forms an antibonding in-plane electron arrangement. It is noteworthy that due to the C_*s*_ symmetry the SONO is distorted and the electron cloud on the left is larger than that on the right as shown in the middle panel of Figure 2. In addition, its carbon ring is narrower than the rings in the other two off-center structures. Such strong distortion of the ring in structure (d) may increase the energy of the system substantially as it increases the ring strain. Nevertheless, the structure may still be energy favored, because the total energy of the system is lowered by the resulting stronger electrostatic attraction between the negatively charged ring and the Li^+^ cation. The combination of both effects results in this low-symmetry structure (d).

The question arises: Which structure is the global minimum among the structures (c), and (d)? We will attempt to provide an answer in the next subsection.

### C The Low-Lying Electronic States of Li@*C*_16_ *in Different Structures*

**FIGURE 3:**
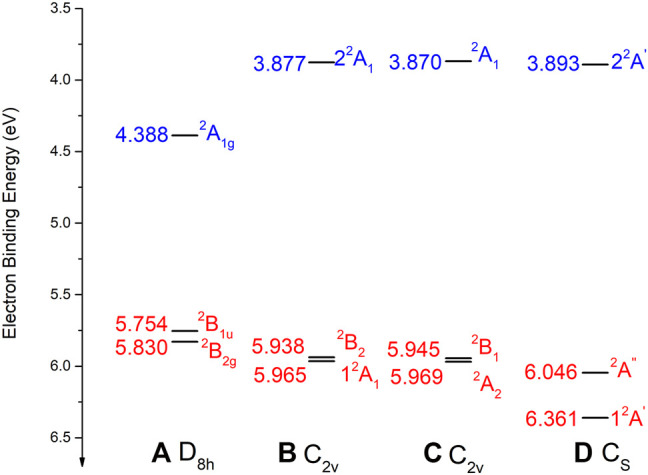
The binding energies of the low-lying electronic states of Li@C_**16**_ obtained at the EA-EOM-CCSD level of theory at the optimized geometries of the respective structures **(A–D)**. For the structures see Figure 1 The data of Li@C_**16**_ in D_**8*h***_ are taken from (Yang et al., 2019b). For more details see text. Charge-separated states are shown in red and non-charge-separated (encircled-electron) states are colored in blue.

The low-lying electronic states of Li@C_16_ have been computed for all the structures discussed above at the EA-EOM-CCSD level of theory. The resulting EBEs, i.e., the electron binding energies gained by adding an electron to the closed-shell Li^+^@C16 cation at the respective geometry, are collected in Figure 3. As already investigated in our previous study (Yang and Cederbaum, 2019b), there are two kinds of electronic states in all Li@C_*n*_ endocircular systems. Charge-separated states as discussed above and non-charge-separated states called encircled-electron states where Li and the carbon ring are both neutral. In larger rings the latter states can be rather low-lying in energy. This is not the case for the still too small ring in Li@C_16_.

As seen in Figure 3, all the low-lying charge-separated states of the off-center structures (b), (c) and (d) possess higher binding energies than the at-center structure of D_8*h*_ symmetry and these EBEs grow with the distortion of the ring. This trend is obviously due to the growing electrostatic interaction of Li^+^ and the negatively charged ring. In contrast to this trend of the charge-separated states, the EBEs of the non-charge-separated encircled-electron states of these species decrease in value along the same structures. This finding is in accord with the fact that the EBEs of the latter states are largest for highly symmetric structures (Yang and Cederbaum, 2021b) and thus decrease the more the SONOs are distorted by the off-center cation.

It is noteworthy that the EBEs of the electronic ground states of structures (b) and (c) are similar in value. Moreover, the two lowest-lying states are quasi-degenerate as their relative energy roughly amounts to only 0.03 eV. The structure (c) is slightly energy favored. The electronic ground state of structure (d) possesses the highest EBE among the found structures. Its electronic ground state is lower in energy than its first excited state by 0.32 eV. Clearly, the highly distorted ring geometry has a stronger influence on state lying in the plane than on the one vertical to the plane.

The total energies of the electronic ground and excited states of all structures investigated are collected in Table 2. These energies have been computed on the EA-EOM-CCSD level at the optimized geometries reported above. As can be seen, the ground state energies of all the structures with off-center Li^+^ are rather close to each other. According to the present calculations, the ground state of structure (c) is lowest in energy among all the species, but the electronic ground state potential energy surface connecting all the computed structures is rather flat. The ground state energy of (b) is nearly the same as that of (c) and that of (d) is 0.1 eV higher than that of (c). However, we have to remember that in contrast to the structures (a), (b), and (c) which have been optimized on the EA-EOM-CCSD level, the geometry of (d) has been optimized due to cost reasons on the DFT level. On the DFT level, (d) is the global minimum and it is seen from the EA-EOM-CCSD data in Table 2 that either this is incorrect or the DFT geometry is rather imprecise so that the above mentioned 0.1 eV can be overcompensated by a geometry shift. Indeed, for the C_2*v*_ structures (b) and (c) it has been found that the DFT calculations underestimate the overall distortion (see Section A.2) and this can also be expected for structure (d). The question which of the structures (c) or (d) relates to the global minimum still remains open.

**TABLE 2:** The total and relative energies of the low-lying states of the structures (a) to (d) of Li@C_16_ shown in Figure 1, computed at the EA-EOM-CCSD level of theory at the optimized geometries reported [i.e., optimized *via* EA-EOM-CCSD for structures (a), (b) and (c) and *via* DFT for structure (d)]. Also shown are the relative energies of the ground states of structures (a) to (d) calculated at the DFT level of theory at the geometries optimized on the DFT level. The data of structure (a) are taken from (Yang and Cederbaum, 2021b).

	Structure (a)			Structure (b)		
	^2^B_2*g*_	^2^B_1*u*_	^2^A_1*g*_	^2^B_2_	1^2^A_1_	2^2^A_1_
Total energy (a.u.)	−615.562 645	−615.559 854	−615.509 658	−615.580 837	−615.579 862	−615.504 114
Relative energy at EA-EOM-CCSD (eV)	0.499	0.575	1.941	0.004	0.030	2.091
Relative energy at DFT level (eV)	0.354	–	–	0.002	–	–

	Structure (c)			Structure (d)		
	^2^A_2_	^2^B_1_	^2^A_1_	1^2^A′	^2^A″	2^2^A′
Total energy (a.u.)	−615.580 971	−615.580 104	−615.503 831	−615.576 574	−615.564 247	−615.483 727
Relative energy at EA-EOM-CCSD level (eV)	0.000	0.024	2.099	0.120	0.455	2.646
Relative energy at DFT level (eV)	0.000	–	–	−0.033	–	–

## IV Conclusion

Non-covalent interactions, such as in charge-separated donor-acceptor complexes and in non-charge-separated van der waals systems, plays an important role in the field of catalysis. The carbon allotropes accommodating guest atoms have promising applications in this field, since these compounds may possess these two kinds of non-covalent interactions. In this paper we studied the off-center geometry of one of these promising species Li@C_16_.

We have performed an extended search for stationary structures of neutral Li@C_16_ and found that there are three structures with Li^+^ off-center and a negatively charged carbon ring rather close in energy. By employing a state-of-the-art equation-of-motion coupled-cluster method, we have optimized the geometries of two of the three possible off-center structures of the neutral endocircular Li@C_16_ system and due to cost reasons the third, low symmetry structure, has been optimized by a suitable DFT method. All the structures are showing alternating single and triple C-C bond pattern, regardless of the position of Li^+^ cation. Moreover, all the structures are considerably distorted by the off-center Li^+^ cation. The similar energies of the structures and their distortions show that the carbon rings are rather flexible which can be considered as an interesting property of the endocircular systems. Since the energy landscape is shallow and the geometries of energetically nearby structures are very different, one encounters at finite temperature severe large amplitude motion of both the nagetively charged carbon ring and of the Li cation.

In addition to the geometries of the structures we have also calculated the total energies and the binding energies of the low-lying electronic states of the three structures at their respective optimized geometries employing the state-of-the-art equation-of-motion coupled-cluster method. The calculations show that charge-separated as well as encircled-electron states are among the low-lying electronic states of each of the structures. The charge-separated states are favored energetically. The structures are the result of the balance between the ionic attraction of Li^+^ and the negatively charged ring and the energy paid by distorting the ring.

The singly occupied natural orbitals describing the excess electron on the carbon ring in the presence of all other electrons have been computed and analyzed. The analysis sheds light on the different structures found. There is a one to one relationship between such a natural orbital and the structure. This makes clear why several structures close by in energy are to be expected on the electronic potential energy surface.

Until now there are no experimental studies on endocircular Li@C_*n*_ and we hope that the interesting results found will stimulate such studies.

## Data Availability

The original contributions presented in the study are included in the article/Supplementary Material, further inquiries can be directed to the corresponding author.
