# Supplementary material for: On the Endocircular Li@C16 System
Source: Front Chem. 2022 Feb 4;10:813563. doi: 10.3389/fchem.2022.813563 (PMC8854773; doi:10.3389/fchem.2022.813563)
Supplement: Supplementary file 1 [file DataSheet1.pdf]

On the endocircular Li@C_{16} system

Yi-Fan Yang and Lorenz S. Cederbaum[1]

Theoretical Chemistry, Institute of Physical Chemistry, University of Heidelberg, Im Neuenheimer Feld 229, 69118 Heidelberg

Table of Contents

Table S1. Cartesian coordinates (EA-EOM-CCSD/cc-pVTZ). 2

Table S2. Cartesian coordinates (ωB97XD/cc-pVTZ)..4

Table S3. Vibrational frequencies (EA-EOM-CCSD/cc-pVTZ) 6

Table S4. Vibrational frequencies (ωB97XD /cc-pVTZ) 8

[1] Corresponding author. Email: Lorenz.Cederbaum@pci.uni-heidelberg.de

Table S1. Cartesian coordinates of structures (a), (b), and (c) of neutral Li@C_{16} optimized at EA-EOM-CCSD/cc-pVTZ level. The coordinates of the center of mass of the rings are also listed. Units are Å.

Structure (a)	Li	0.00000000	0.00000000	0.00000000
	C	-3.22891004	-0.67265018	0.00000000
	C	-2.75881969	-1.80754868	0.00000000
	C	-1.80754868	-2.75881969	0.00000000
	C	-0.67265018	-3.22891004	0.00000000
	C	0.67265018	-3.22891004	0.00000000
	C	1.80754868	-2.75881969	0.00000000
	C	2.75881969	-1.80754868	0.00000000
	C	3.22891004	-0.67265018	0.00000000
	C	3.22891004	0.67265018	0.00000000
	C	2.75881969	1.80754868	0.00000000
	C	1.80754868	2.75881969	0.00000000
	C	0.67265018	3.22891004	0.00000000
	C	-0.67265018	3.22891004	0.00000000
	C	-1.80754868	2.75881969	0.00000000
	C	-2.75881969	1.80754868	0.00000000
	C	-3.22891004	0.67265018	0.00000000
Center of (a)		0.00000000	0.00000000	0.00000000
Structure (b)	Li	0.00000000	1.31883787	0.00000000
	C	0.67453374	3.37161613	0.00000000
	C	-0.67453374	3.37161613	0.00000000
	C	1.76221397	2.77954072	0.00000000
	C	-1.76221397	2.77954072	0.00000000
	C	2.64695553	1.76842298	0.00000000
	C	-2.64695553	1.76842298	0.00000000
	C	3.06119811	0.60905558	0.00000000
	C	-3.06119811	0.60905558	0.00000000
	C	3.11090651	-0.73578468	0.00000000
	C	-3.11090651	-0.73578468	0.00000000
	C	2.68771320	-1.88596178	0.00000000
	C	-2.68771320	-1.88596178	0.00000000
	C	1.79341886	-2.89720111	0.00000000
	C	-1.79341886	-2.89720111	0.00000000
	C	0.67568768	-3.39522828	0.00000000
	C	-0.67568768	-3.39522828	0.00000000
Center of (b)		0.00000000	-0.04819256	0.00000000

Table S1. (continued)

Structure (c)	Li	0.00000000	1.33658194	0.00000000
	C	-0.61987357	3.39820764	0.00000000
	C	0.61987357	3.39820764	0.00000000
	C	-1.81315851	2.77508091	0.00000000
	C	1.81315851	2.77508091	0.00000000
	C	-2.57283514	1.80084270	0.00000000
	C	2.57283514	1.80084270	0.00000000
	C	-3.06963398	0.55198453	0.00000000
	C	3.06963398	0.55198453	0.00000000
	C	-3.08932657	-0.67535565	0.00000000
	C	3.08932657	-0.67535565	0.00000000
	C	-2.66042114	-1.95354700	0.00000000
	C	2.66042114	-1.95354700	0.00000000
	C	-1.83816281	-2.86049288	0.00000000
	C	1.83816281	-2.86049288	0.00000000
	C	-0.61165109	-3.42744787	0.00000000
	C	0.61165109	-3.42744787	0.00000000
Center of (c)		0.00000000	-0.04884095	0.00000000

Table S2. Cartesian coordinates of structures (a), (b), (c) and (d) of neutral Li@C_{16} optimized at ωB97XD/cc-pVTZ level. The coordinates of the center of mass of the rings are also listed. Units are Å.

Structure (a)	Li	0.00000000	0.00000000	0.00000000
	C	0.61434700	3.23366000	0.00000000
	C	-0.61434700	3.23366000	0.00000000
	C	-1.85213398	2.72095184	0.00000000
	C	-2.72095184	1.85213398	0.00000000
	C	-3.23366000	0.61434700	0.00000000
	C	-3.23366000	-0.61434700	0.00000000
	C	-2.72095184	-1.85213398	0.00000000
	C	-1.85213398	-2.72095184	0.00000000
	C	-0.61434700	-3.23366000	0.00000000
	C	0.61434700	-3.23366000	0.00000000
	C	1.85213398	-2.72095184	0.00000000
	C	2.72095184	-1.85213398	0.00000000
	C	3.23366000	-0.61434700	0.00000000
	C	3.23366000	0.61434700	0.00000000
	C	2.72095184	1.85213398	0.00000000
	C	1.85213398	2.72095184	0.00000000
Center of (a)		0.00000000	0.00000000	0.00000000
Structure (b)	Li	0.00000000	1.19961600	0.00000000
	C	-0.67273100	-3.38170700	0.00000000
	C	-1.79078200	-2.88283600	0.00000000
	C	-2.67361700	-1.86953500	0.00000000
	C	-3.10571700	-0.72149700	0.00000000
	C	-3.05176100	0.61812200	0.00000000
	C	-2.65687000	1.78562300	0.00000000
	C	-1.76800800	2.78825800	0.00000000
	C	-0.67156000	3.36366800	0.00000000
	C	0.67156000	3.36366800	0.00000000
	C	1.76800800	2.78825800	0.00000000
	C	2.65687000	1.78562300	0.00000000
	C	3.05176100	0.61812200	0.00000000
	C	3.10571700	-0.72149700	0.00000000
	C	2.67361700	-1.86953500	0.00000000
	C	1.79078200	-2.88283600	0.00000000
	C	0.67273100	-3.38170700	0.00000000
Center of (b)		0.00000000	-0.03748800	0.00000000

Table S2. (Continued)

Structure (c)	Li	0.00000000	1.20597200	0.00000000
	C	0.61202200	-3.40484300	0.00000000
	C	-0.61202200	-3.40484300	0.00000000
	C	-1.83130000	-2.83683100	0.00000000
	C	-2.65993300	-1.93448300	0.00000000
	C	-3.08465200	-0.66098500	0.00000000
	C	-3.08791400	0.56799000	0.00000000
	C	-2.58892300	1.81126100	0.00000000
	C	-1.82319300	2.78184500	0.00000000
	C	-0.61944300	3.37455300	0.00000000
	C	0.61944300	3.37455300	0.00000000
	C	1.82319300	2.78184500	0.00000000
	C	2.58892300	1.81126100	0.00000000
	C	3.08791400	0.56799000	0.00000000
	C	3.08465200	-0.66098500	0.00000000
	C	2.65993300	-1.93448300	0.00000000
	C	1.83130000	-2.83683100	0.00000000
Center of (c)		0.00000000	-0.03768663	0.00000000
Structure (d)	Li	-0.05930214	1.22397617	0.00000000
	C	-2.93546927	-0.26810884	0.00000000
	C	-2.84182264	-1.50553214	0.00000000
	C	-2.06974089	-2.59913807	0.00000000
	C	-1.14255598	-3.40513332	0.00000000
	C	0.20697833	-3.51479373	0.00000000
	C	1.39663774	-3.24663702	0.00000000
	C	2.35522813	-2.28169885	0.00000000
	C	2.89750288	-1.19448394	0.00000000
	C	2.98055950	0.16430361	0.00000000
	C	2.70149119	1.34808835	0.00000000
	C	2.02882804	2.53453475	0.00000000
	C	1.03381317	3.24782266	0.00000000
	C	-0.23608619	3.75640988	0.00000000
	C	-1.33070321	3.12915144	0.00000000
	C	-2.24451413	2.18053324	0.00000000
	C	-2.77049559	1.04269388	0.00000000
Center of (d)		0.00185319	-0.03824926	0.00000000

Table S3. Vibrational frequencies of the optimized structures (a), (b), and (c) of neutral Li@C_{16}. These structures are shown in Table S1. The Frequencies are obtained at EA-EOM-CCSD/cc-pVTZ level. Imaginary frequencies are marked in bold. All units are in cm^{-1}.

Li@C_{16} (a)		Li@C_{16} (b)		Li@C_{16} (c)	
**E1u**	**199.26i**	**B1**	**32.58i**	B2	34.2598
**E1u**	**199.26i**	B2	50.94	B1	34.4112
**E2g**	**27.45i**	A1	67.68	A1	70.8967
**E2g**	**27.45i**	A2	97.99	A2	98.6607
E2u	92.54	B2	100.34	B2	100.614
E2u	92.54	B1	115.40	B1	116.719
E3u	148.09	A1	197.43	A1	195.758
E3u	148.09	A2	221.22	B2	220.333
A2u	150.29	B2	227.23	A2	226.598
E3g	218.74	B1	237.23	B1	228.832
E3g	218.74	A1	340.27	A1	342.331
B2g	310.18	A2	351.93	B2	344.89
A2g	313.12	B2	367.71	B1	354.723
B1g	328.78	B1	373.98	A2	365.527
B1u	346.48	A1	381.65	A1	397.816
B2u	358.21	B1	465.50	B1	472.512
E3u	471.79	B1	488.59	A1	487.88
E3u	471.79	A1	491.07	A2	488.448
E3g	489.73	A2	494.14	B2	493.12
E3g	489.73	B2	498.40	B1	493.685
A1g	498.45	A1	511.42	A1	507.217
E1u	544.78	B1	573.38	B1	571.993
E1u	544.78	A1	575.11	A1	576.606
E2g	557.30	B1	607.02	A1	607.195
E2g	557.30	A1	613.70	B1	609.168
E2u	638.28	A2	634.53	A2	632.752
E2u	638.28	B1	652.15	B1	649.146
E1u	674.01	B2	671.53	B2	671.459
E1u	674.01	A1	697.58	A1	700.802
E1g	771.54	A2	801.46	A2	806.018
E1g	771.54	B2	862.53	B2	871.061
A1u	774.65	A2	957.81	A2	974.596
E2g	1020.23	B1	1015.58	B1	1013.69
E2g	1020.23	A1	1022.92	A1	1025.74
E3u	1339.32	A1	1338.91	B1	1337.16

Table S3. (continued)

$Li@C_{16}$ (a)		$Li@C_{16}$ (b)		$Li@C_{16}$ (c)	
E3u	1339.32	B1	1343.63	A1	1346.49
B1g	1514.85	A1	1511.72	B1	1511.45
B2g	2053.42	B1	2038.28	A1	2036.42
E1u	2091.19	B1	2074.33	B1	2073.37
E1u	2091.19	A1	2076.19	A1	2078.94
E3u	2153.17	B1	2149.46	B1	2150.07
E3u	2153.17	A1	2149.95	A1	2150.59
E2g	2245.99	A1	2237.98	A1	2237.16
E2g	2245.99	B1	2242.64	B1	2242.76
A1g	2267.42	A1	2250.21	A1	2251.79

Table S4. Vibrational frequencies of optimized structures (a), (b), (c), and (d) as in Table 3. These structures are shown in Table S2. The Frequencies are obtained at ωB97XD/cc-pVTZ level. The frequency calculation of structure (d) is done without symmetry restriction. Imaginary frequencies are marked in bold. All units are in cm^{-1}. Zero-point energy corrections of structure (c) and (d) are 17098.44 and 16958.74 cm^{-1}, respectively.

**Li@C_{16} (a)**		**Li@C_{16} (b)**		**Li@C_{16} (c)**		**Li@C_{16} (d)**
**E1u**	**206.41i**	**B1**	**37.82i**	B2	21.83	33.46
**E1u**	**206.41i**	B2	61.97	B1	57.17	43.48
**E2g**	**29.00i**	A1	66.18	A1	68.72	84.06
**E2g**	**29.00i**	A2	90.54	A2	91.41	87.43
E2u	83.45	B2	95.18	B1	94.15	118.75
E2u	83.45	B1	108.99	B2	114.93	143.53
A2u	145.75	A1	196.83	A1	193.13	149.73
E3u	163.43	A2	205.63	B1	205.30	195.48
E3u	163.43	B2	211.53	A2	211.52	209.14
E3g	201.28	B1	217.07	B2	214.34	219.25
E3g	201.28	A1	284.08	A1	277.32	220.27
A2g	244.81	A2	294.46	B2	290.48	260.57
B2u	306.31	B2	315.15	B1	311.05	292.43
B1g	308.59	B1	325.27	A2	326.03	299.06
B1u	319.98	A1	340.30	B2	337.70	309.10
B2g	323.06	B1	349.80	A1	349.66	344.18
A1u	354.59	B1	361.90	B1	365.24	360.28
E3g	394.20	A1	363.14	A2	374.55	400.21
E3g	394.20	A2	388.81	A2	396.76	403.45
E1g	408.77	B2	406.54	B1	413.15	417.26
E1g	408.77	A1	413.07	A2	418.39	453.99
E2u	429.59	B1	443.98	B1	445.85	456.10
E2u	429.59	A1	444.21	A2	449.22	462.47
E3u	447.77	B1	459.94	A1	461.24	469.86
E3u	447.77	A1	467.76	B2	467.14	477.93
E1u	484.96	A2	487.33	A1	486.62	488.39
E1u	484.96	B1	510.69	B2	512.03	512.83
A1g	505.37	B2	521.13	A1	520.41	522.26
E2g	516.59	A1	533.77	B2	534.00	529.43
E2g	516.59	A2	541.58	A1	541.41	532.20
E1u	684.29	B2	662.07	B2	661.20	638.77
E1u	684.29	A2	706.58	A1	707.11	725.30

Table S4. (continued)

Li@C_{16} (a)		Li@C_{16} (b)		Li@C_{16} (c)		Li@C_{16} (d)
E2g	1046.51	B1	1039.25	B2	1037.74	1008.35
E2g	1046.51	A1	1043.10	A1	1044.65	1037.86
E3u	1388.60	A1	1376.60	B2	1376.13	1327.62
E3u	1388.60	B1	1380.56	A1	1380.41	1358.85
B2g	1563.96	A1	1551.82	B2	1550.97	1509.52
E1u	1946.96	B1	1912.59	B2	1912.76	1905.93
E1u	1946.96	B1	1958.95	A1	1957.6	1914.26
B1g	2064.15	A1	2046.83	A1	2047.56	2038.16
E3u	2158.60	B1	2143.50	B2	2143.78	2113.65
E3u	2158.60	A1	2148.90	A1	2149.03	2151.18
E2g	2240.25	A1	2219.15	A1	2218.3	2182.24
E2g	2240.25	B1	2229.29	B2	2228.93	2244.29
A1g	2244.45	A1	2229.52	A1	2229.95	2264.93
